# Enhancing Stability of Camelid and Shark Single Domain Antibodies: An Overview

**DOI:** 10.3389/fimmu.2017.00865

**Published:** 2017-07-25

**Authors:** Ellen R. Goldman, Jinny L. Liu, Dan Zabetakis, George P. Anderson

**Affiliations:** ^1^Center for BioMolecular Science and Engineering, US Naval Research Laboratory, Washington, DC, United States

**Keywords:** single domain antibody, camelid, shark, protein engineering, stability, melting temperature, refolding

## Abstract

Single domain antibodies (sdAbs) are gaining a reputation as superior recognition elements as they combine the advantages of the specificity and affinity found in conventional antibodies with high stability and solubility. Melting temperatures (*T*_m_s) of sdAbs cover a wide range from below 50 to over 80°C. Many sdAbs have been engineered to increase their *T*_m_, making them stable until exposed to extreme temperatures. SdAbs derived from the variable heavy chains of camelid and shark heavy chain-only antibodies are termed VHH and VNAR, respectively, and generally exhibit some ability to refold and bind antigen after heat denaturation. This ability to refold varies from 0 to 100% and is a property dependent on both intrinsic factors of the sdAb and extrinsic conditions such as the sample buffer ionic strength, pH, and sdAb concentration. SdAbs have also been engineered to increase their solubility and refolding ability, which enable them to function even after exposure to temperatures that exceed their melting point. In addition, efforts to improve their stability at extreme pH and in the presence of chemical denaturants or proteases have been undertaken. Multiple routes have been employed to engineer sdAbs with these enhanced stabilities. The methods utilized to achieve these goals include grafting complementarity-determining regions onto stable frameworks, introduction of non-canonical disulfide bonds, random mutagenesis combined with stringent selection, point mutations such as inclusion of negative charges, and genetic fusions. Increases of up to 20°C have been realized, pushing the *T*_m_ of some sdAbs to over 90°C. Herein, we present an overview of the work done to stabilize sdAbs derived from camelids and sharks. Utilizing these various strategies sdAbs have been stabilized without significantly compromising their affinity, thereby providing superior reagents for detection, diagnostic, and therapeutic applications.

## Introduction

Single domain antibodies (sdAbs) are recombinant autonomous variable domains with antigen-binding functionality. The first reported sdAbs were variable heavy domains (VH) derived from IgGs (1). In this pioneering work, several mouse-derived VHs with specificity for lysozyme were shown to have affinities in the 19–27 nM range; however, they were described as “relatively sticky.” The idea of a single domain, antibody-derived binding unit, however, was appealing as it offered potential advantages over large intact antibodies and even over Fv fragments containing paired VH and variable light (VL) domains.

In the early 1990s, the first report on the discovery of heavy chain-only antibodies (hcAbs) in camelids was published (2). These unique antibodies were heavy chain dimers, completely lacking light chains. They also lacked the first IgG constant domain, CH1. Consequently, their antigen-binding regions consist of one single VH domain termed VHH. It was observed that in VHHs, several framework region (FR) positions located in the area that would have formed the interface with the VL in conventional tetrameric IgGs were altered relative to the VH consensus sequence, which resulted in a more hydrophilic surface overall. In general, however, the VHH is closely related to the VH domain of conventional IgG. As early as 1994, it was observed that a human VH showed a decrease in aggregation when it was “camelized” by substituting three key FR residues in its former interface with those found in VHHs at equivalent positions (3). In 1995, a report detailed hcAbs derived from shark (IgNARs) whose variable domains, termed VNARs, are more closely related to T-cell receptors than IgG (4).

Production of the first VHHs was described in 1997, and these were demonstrated to function after extensive heating at 37°C (5). Likewise VNARs, described a few years after VHHs, showed an ability to bind antigen after heat challenge (6, 7). This work was key in showing that sdAbs have the potential to provide recognition reagents that combine the specificity and affinity of natural antibodies with high stability and solubility. Because of their stability and solubility, sdAbs including VHHs and VNARs as well as VHs and VLs engineered from conventional antibody variable domains, are being exploited for a number of applications ranging from therapeutics and detection to biotechnology (8–12).

The melting temperatures (*T*_m_s) of sdAbs, which cover a wide range from below 50 to over 80°C, are used as a measure of sdAb stability. However, sdAb stability is also defined by their ability to function after heating. Unlike conventional antibodies and recombinant-binding elements derived from paired VH and VL domains which generally lose their binding ability upon heat denaturation due to irreversible aggregation (13), the binding ability of many sdAbs is restored after heating due to their ability to refold. Refolding can be influenced by variables such as the ionic strength and pH of the sample buffer as well as sdAb concentration. Time and temperature in the unfolded state can also impact the ability of sdAbs to refold, as extended time can allow for a slow aggregation process to accumulate, and temperatures near the transition point often appear more damaging as the temperature is low enough to allow interactions to occur but still too high to allow the sdAb to proceed toward its native conformation. Extended heat exposure can also cause chemical alterations that can prevent proper refolding. For example, chemical modification of Asn was found to be detrimental to the ability of an sdAb to refold (14); disulfide shuffling can also negatively impact the refolding process (15). With so many dynamic issues to resolve to achieve successful refolding, it is not surprising that often the preferred solution for sdAb stabilization is to engineer an increased *T*_m_ to prevent denaturation from occurring in the first place.

Recombinant DNA technology enables the manipulation of sdAb genes to increase sdAb *T*_m_s and refolding abilities. Protein engineering has also been utilized to improve sdAb stability against chemical denaturants, extreme pH, and proteases. Mutational approaches to improve the biophysical properties of sdAbs derived from conventional human variable chains (VH and VL) have been detailed in recent reviews (16–18). Herein, we focus on engineering-enhanced stability into sdAbs derived from camelids and sharks (VHHs and VNARs, respectively). A number of reviews detail the properties of sdAbs and their use in biotechnology and therapeutic applications (8, 11, 19–21); however, none has focused on the growing body of work that utilizes protein engineering to improve the stability of VHHs and VNARs. We cover a number of methods that have been successfully employed to increase sdAb stability including grafting complementarity-determining regions (CDRs) onto stable frameworks, introduction of non-canonical disulfide bonds, random mutagenesis combined with stringent selection, point mutations, and genetic fusions (Table 1). Through these strategies, stabilized sdAbs have been developed that retain their binding ability under extreme conditions, thereby providing superior reagents for a myriad of biotechnology, detection, diagnostic, and therapeutic applications.

**Table 1:** Strategies to stabilize VHHs and VNARs.

Strategy	Benefits	Drawbacks
Complementarity-determining region grafting	Can increase stability and expression in *E. coli* (up to 10°C increase in *T*_m_ reported)	– Unavailability of a universal stable framework – Affinity may be compromised
Addition of non-canonical disulfide bond	– Reliable approach to increase *T*_m_s (*T*_m_ increases of ~4–20°C have been reported). – Increased stability at extreme pH and in the presence of denaturants – Imparts increased protease resistance	– Affinity may be compromised – May lead to decreased expression in *E. coli*
Random mutagenesis/Stringent selection	Employed to pull out binding single domain antibodies (sdAbs) with improved *T*_m_s and protease stability from sdAb display libraries	Needs to be done separately for each sdAb/target
Point mutations	– Improved refolding ability – Increased function after heating – Increased *T*_m_ (*T*_m_ increases of ~3–9°C have been reported)	– Need to tailor to each sdAb sequence (might not be universal) – Some mutations that improve refolding ability may decrease *T*_m_
Fusions	– Universal construct that does not have to be engineered for each sdAb sequence – Improved refolding ability (in the absence of canonical disulfide bond has been reported)	– May lead to decrease in *T*_m_ – May lead to decrease in expression yield in *E. coli*

## Analytical Techniques for Studying sdAb Stability

**Figure 1:**
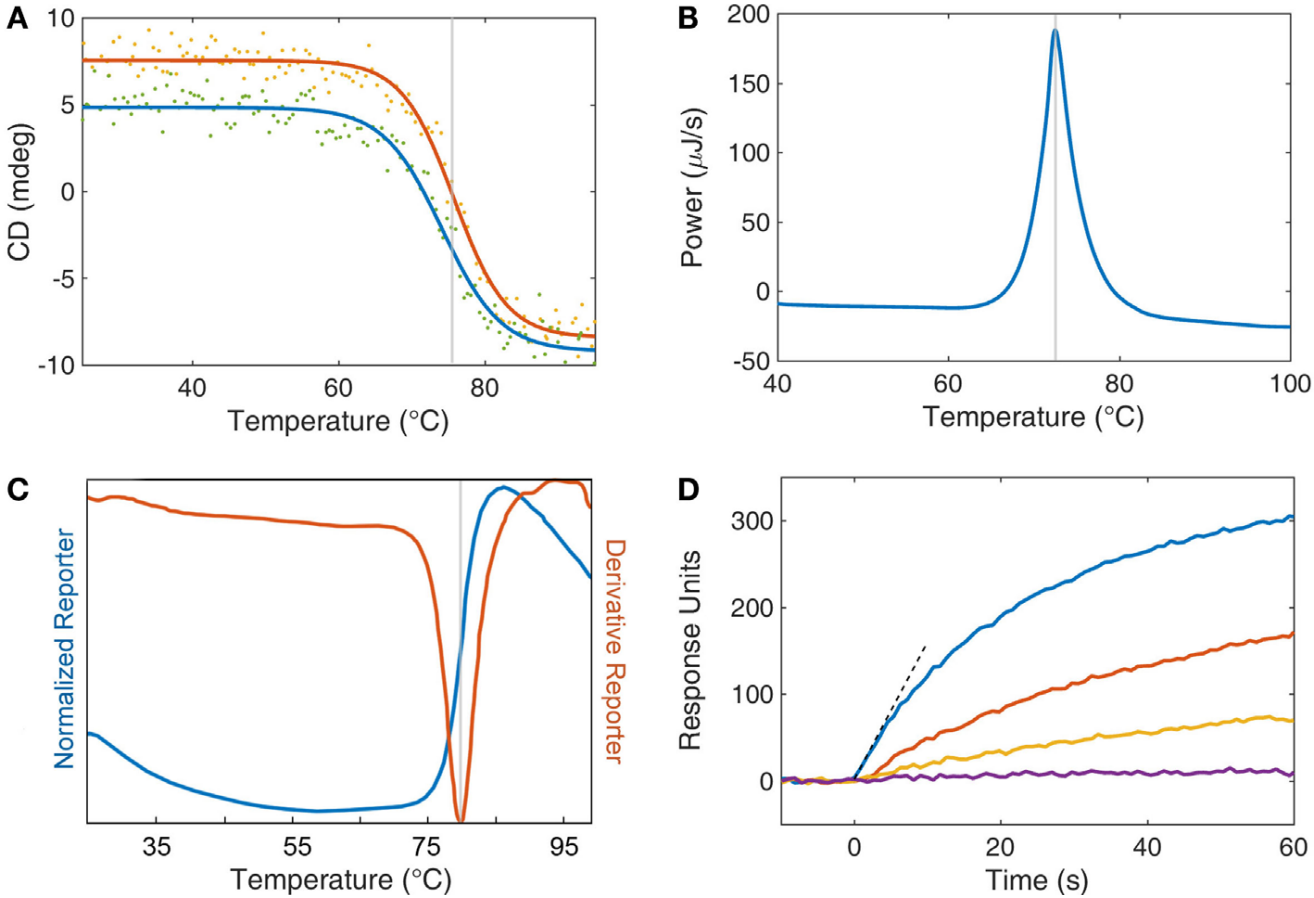
Analytical techniques for studying single domain antibody stability. **(A)** Circular dichroism (CD) is displayed as a function of temperature for both heating (red) and cooling (blue). The inflection point of the heating curve is reported as the *T*_m_ of the protein (gray line). Refolding after thermal denaturation is determined by the recovery of CD signal upon cooling. **(B)** Differential scanning calorimetry compares the thermodynamics of a protein sample with a buffer reference while undergoing heating. Protein unfolding is an enthalpic process and so an energy input is required to retain equilibrium with a buffer control. This energy input is recorded (blue) and the peak is reported (gray) as the *T*_m_. **(C)** A thermal dye-based assay can be employed whereby a fluorescent dye’s quantum yield is enhanced by the interaction of the dye with hydrophobic amino acids upon denaturation of the protein. Fluorescent data are collected as both a direct measurement (normalized reporter, blue curve) and the first derivative of the fluorescence (red curve). The *T*_m_ of the protein (gray line) is taken as the peak of the derivative reporter. **(D)** Surface plasmon resonance is used as an activity assay to assess the retention of function after heat treatment. Individual protein samples are subjected to increasing levels of thermal stress (blue, red, orange, and purple) and then returned to room temperature. The antibodies are then exposed to an antigen-coated surface, and the response to binding is recorded as response units (also called resonance units). The initial rate (dashed line) is a measure of function and may be compared with a control sample to determine loss or retention of function.

There are a number of techniques that researchers have utilized to evaluate the biophysical characteristics of sdAbs. Methods for determining stability can be broken broadly into those that measure a physical parameter and those that assess the ability of an sdAb to bind antigen after heating. While circular dichroism, differential scanning calorimetry, and the protein thermal shift method all measure *T*_m_, each approach interrogates a very different biophysical property. Nonetheless, they all track fairly close together, typically within ±3°C. In this section, we will discuss the methods utilized to measure *T*_m_ as well as delve into other critical parameters to ascertain for a stabilized sdAb such as solubility and retention of activity. Several methods used to measure *T*_m_ can also be adapted to measure the stability of sdAbs in the presence of chemical denaturants (22). Examples of data from several of the methods to assess stability, which is discussed subsequently, are shown in Figure 1.

### Circular Dichroism

Circular dichroism (CD) is one of the most commonly utilized methods to track protein unfolding as the far UV CD reflects a protein’s secondary structure (23). This technique is used to assess both the *T*_m_ and refolding ability of sdAbs. CD, which measures the differential absorption of left-handed and right-handed circularly polarized light can monitor the folding status of a protein. As CD is typically performed at relatively low protein concentrations (<0.1 mg/mL), sdAbs can typically be observed to refold as the sample is cooled. For many sdAbs, it is possible to repeat this process many times generating successive unfolding and refolding curves for the same sample. However, not every sdAb isolated is observed to refold in these experiments. In these cases, it would appear that those sdAbs had a greater propensity to aggregate when in the unfolded state. It is this propensity to aggregate that limits the ability of most multidomain proteins, such as scFvs, to refold following thermal denaturation. In our experience, we have found the unfolding and refolding transition of sdAbs to be a rapid process that occurs at virtually the same temperature (24).

### Differential Scanning Calorimetry

Differential scanning calorimetry (DSC) is also a popular method for measuring the melting temperatures of proteins (25). DSC measures the amount of energy required to raise the temperature of protein sample to the same extent as a buffer control. The additional energy consumed as a protein unfolds marks the *T*_m_ transition. As the amount of energy consumed to unfold a single molecule is very small, DSC requires large amounts of protein to generate a robust signal, ideally 3–5 mg. Another drawback of DSC is that at least partly due to the high concentration of protein required to make the measurement, refolding is not often observed. However, unlike CD, DSC can be performed in various buffers, it can also be useful in determining the *T*_m_ of sdAbs with *T*_m_s above 90°C, as well as corroborate values obtained through other methods.

### Protein Thermal Shift Assay

Protein thermal shift assay, also referred to as a fluorescent dye-based melting assay or dye melt assay, monitors the extrinsic fluorescence of an organic fluorophore, such as Sypro orange, added to the protein solution prior to heating. This method is an attractive approach, due to both the small amount of protein required (5–10 µg) and the ability to test multiple samples simultaneously, as the measurement is performed using a real time PCR instrument (26, 27). Here, the fluorescence intensity of the Sypro orange dye increases rapidly as the protein unfolds and the dye can associate with the hydrophobic amino acids normally inaccessible in the protein’s core. Typically, the negative of the first derivative of the fluorescence intensity versus temperature is plotted with the dip in the plot representing the *T*_m_. The main limitation of this method is that unlike CD one cannot evaluate refolding.

### Intrinsic Fluorescence

Intrinsic fluorescence can also be monitored during the heating process, as the quantum yield of the buried hydrophobic residues, tryptophan and to a less extent tyrosine, will change upon loss of the proteins secondary structure (28). Some CD instruments are equipped to simultaneously measure intrinsic fluorescence. In one study examining the *T*_m_ of an sdAb by these two methods, it appeared that changes in intrinsic fluorescence were not tightly aligned with loss in secondary structure, suggesting that monitoring intrinsic fluorescence should be considered only as a secondary method (29).

### Activity Assays

While the methods described previously directly measure the temperature at which the protein loses its secondary structure, it is not uncommon for direct binding activity measurements to be performed to monitor the stability of the sdAb following exposure to temperature extremes or harsh conditions. Measuring activity of sdAbs after exposure to harsh conditions is of utmost importance as in some cases CD has shown ~80–90% refolding while the percentage of binding activity after heating was found to be closer to 50% (22). While activity is certainly the gold standard by which any antibody must be measured, certain precautions are necessary to assure that one does not use an excess amount of antibody so as to accurately measure the true percentage of activity remaining.

A number of binding assays can be adapted to measure the ability of sdAbs to function after exposure to elevated temperature. Traditionally, this was assessed by ELISA or similar binding assays (5, 7, 30). Regardless of the assay format, it is important to utilize a sub-saturating concentration of sdAb in order that measured binding is responsive to loss in activity. A pitfall particularly for high affinity antibodies is that depending on the amount of sdAb used in the binding measurements, the assay may not accurately reflect the amount of activity after heat exposure. Ideally, a range of dilutions of the heated sdAb should be assessed.

We have routinely measured the initial binding rate of the sdAb to its target analyte by measuring the signal increase during the first few seconds by surface plasmon resonance. Measuring the initial on-rate has the advantage that one does not require a standard curve and thus is a simple, sensitive, and direct measure of binding activity (31).

The majority of functional characterizations are performed on soluble sdAbs that act as reporters in assays. However, the activity of immobilized sdAbs can also be assessed, for example, by incubating sdAbs immobilized on nitrocellulose at elevated temperatures (32).

### Other Important Parameters

For development of reagents intended for therapeutic applications, other parameters are also of importance, such as proteolytic stability, solubility, and producibility. While it has been observed that proteolytic stability and thermal stability seem to have a positive correlation, the same cannot be said for the other parameters. It has been observed that solubility is enhanced as the net charge on the sdAb is increased, thus increasing intermolecular repulsion, however, the final formulation would also need to take into consideration the role that additives may play in maintaining good solubility. Methods for assessing these other parameters have been reviewed in the context of conventional antibodies (33).

## Stabilizing VHHs

It has been over two decades since the first description of VHHs, and there is now a large body of literature describing VHHs that recognize a wide variety of targets, their properties, engineering, and use in applications from biosensors, to therapeutics, and chaperones for crystallization. Although many VHHs are inherently stable and able to refold, several studies have been geared toward understanding the mechanism of VHH stability and increasing their stability. An overview of several key strategies that have been successfully used to increase VHH stability are given in the following sections and summarized in Table 1.

We have used the IMGT numbering scheme of V domains (34). The antigen receptor numbering and receptor classification tool was used for numbering the amino acid sequences of the VHH (35).

### Complementarity Determining Region Grafting

**Figure 2:**
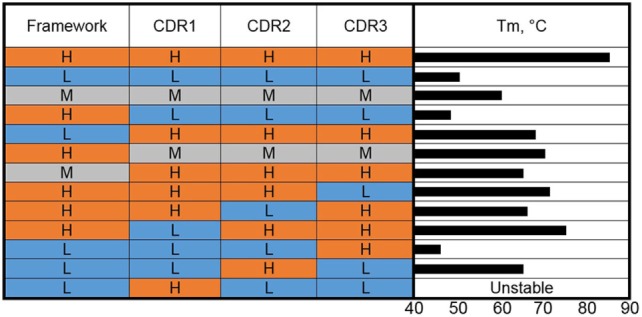
Complementarity-determining region (CDR) grafting and thermal stability. VHHs of high (H), medium (M), and low (L) thermal stability (color coded as red, gray, and blue) were used as the basis for construction of genetic hybrids. The framework regions (as a unit) and the individual CDRs were mixed in various combinations. The *T*_m_s of the resulting hybrids are shown as a bar graph on the right side.

Given the structure of sdAbs, where four FRs are interspersed with three well-defined CDRs, it is a natural approach in antibody engineering to construct hybrid swaps of CDRs and FRs (Figure 2). CDR grafting involves substituting the binding loops that comprise the CDRs of one V domain onto the FR of a different V domain. Taking a lead from the extensive use of CDR grafting to produce humanized murine antibodies, Saerens et al. (36) sought a universal VHH framework that had a set of beneficial defined properties. Their candidate, the cAbBCII10 VHH, featured good expression levels and stability and could be produced in *Escherichia coli* within the reducing environment of the cytoplasm or without its conserved canonical disulfide bond. This VHH was found to function well as a scaffold, retaining the function of grafted CDRs from a donor VHH. The cAbBCII10 VHH was derived from a dromedary and is a member of the dromedary subfamily 2. CDR grafts of subfamily 2 dromedary VHHs onto the cAbBCII10 FR all retained binding and showed increased stability and production at least equivalent to the donor VHH. However, while cAbBCII10 showed promise as a universal scaffold for one large subfamily of dromedary VHHs, it was not ideal for a different subfamily of dromedary VHHs or a llama-derived VHH. This work suggests that it may not be possible to identify one universal VHH scaffold for increased stability but that there is benefit in exploring CDR grafting for improving sdAb properties.

This work of Saerens et al. (36) was extended to humanization of cAbBCII10 for use in the development of human therapeutics (37). A humanized version of the VHH showed a slightly lower *T*_m_ than the wild type (74.3 versus 77.5°C). The humanized version, however, showed only ~8% refolding after heat denaturation. Grafting of CDRs from a dromedary subfamily 2 VHH with a *T*_m_ of 79.7°C and over 90% refolding ability onto the humanized cAbBCII10 scaffold resulted in a VHH that melted at 82.1°C and refolded at 68%, indicating that the CDRs contribute to the stability and refolding ability of VHH either by forming more or less favorable interactions with backbone residues or intra-/inter-CDR interactions.

We carried out a molecular dissection of llama-derived VHHs of high, low, and moderate stability in an effort to reveal the features most responsible for VHH stability (38) (Figure 2). The high stability VHH was specific for Staphylococcal enterotoxin B (SEB), while the other two VHH utilized in these experiments bound ricin. In a first set of experiments, the three CDRs and the FRs of VHHs that melt at 85, 60, and 50°C were exchanged, examining clones resulting from the high melting VHH CDRs on the moderate and low melting FRs and the CDRs from the low and moderate VHHs on the high melting FR. The resultant clones were examined in terms of *T*_m_ and binding kinetics. In each case, grafting the three CDRs from the high melting VHH resulted in VHHs with higher *T*_m_s than those of the FR donors but lower than the CDR donor. Grafting the three CDRs from the medium melting VHH onto the stable FR led to a VHH that showed an increase in *T*_m_ over the CDR donor of 10°C and maintained sub nM affinity (0.46 nM for the graft versus 0.16 nM for the original). The graft variant also showed greater stability than the CDR donor at pH 4.5 (39). Interestingly, grafting the three CDRs from the low melting VHH onto the FR from the high melting clone resulted in a VHH with a low melting point (38). This is another example that while CDR grafting can be an excellent tool to increase the stability of sdAbs, no universal framework has been defined. It also shows how both the CDR and FR sequence contribute to VHH stability.

To further dissect the situation, CDRs from VHHs that melt at 85 and 50°C were mixed-and-matched (38). The results of grafting individual CDRs show that CDR2 was the major contributor to stability in the high *T*_m_ VHH. Grafting only CDR2 from the high melting VHH onto the low melting VHH produced a clone that bound SEB, the target of the high melting VHH, and had a *T*_m_ of 65°C. This work corroborates that both the FR and CDR can be important for stability and that in at least some instances affinity and stability are linked and cannot be freely engineered.

### Introduction of Non-Canonical Disulfide Bonds

The stability of the sdAb has been found to be highly dependent upon the formation of its highly conserved disulfide bond. When VHHs are produced in the *E. coli* cytoplasm, which has a relatively reducing environment, the resultant VHHs are found to have a much lower *T*_m_ than the same VHHs produced in the oxidizing environment of the *E. coli* periplasm (40–42). This difference in stability is attributable to the failure of the conserved disulfide bond to form. Thus, nature has shown a simple and direct method for engineering sdAbs with enhanced stability; namely, the addition of a second disulfide bond. Indeed, engineering of an additional disulfide bond into VHHs is a well-known approach for enhancing their thermal stability (41, 43–47).

Although this technique had been applied to many proteins, it was Hagihara et al. (41) who first showed that it was possible to stabilize VHHs by inserting an extra disulfide bond by changing the highly conserved buried residues Ala54 and Ile78 [IMGT numbering scheme (34)] both to Cys (41). This new disulfide bond adds an additional constraint between two of the β-sheet strands of the sdAb’s secondary structure, resulting in a stabilized tertiary structure, with a *T*_m_ increase of ~10°C over the wild-type sdAb.

Shortly after, Saerens et al. (45) evaluated a VHH that naturally had an extra pair of Cys at the same positions (54/78), leading to a disulfide bond linking FR2 and FR3 (45). They found that the addition of this extra disulfide bond to three additional VHHs resulted in a 4–18°C increase in *T*_m_, however one of the VHHs showed a ~43-fold loss in binding activity. They also examined another location for insertion of an additional disulfide bond that linked FR2 to FR3 by introducing Cys at positions 39 and 87. This location was selected for its distance in the crystal structure of β-strands from opposing β-sheets and with side chains oriented suitably for disulfide bond formation. Testing three different VHHs that included nearly all the possible permutations of these three disulfide bonds, including none at all, they deduced that the native highly conserved disulfide bond was the most favorable, with addition of a second disulfide bond adding further stability but with possible negative impacts on affinity that were not easily predictable.

**Figure 3:**
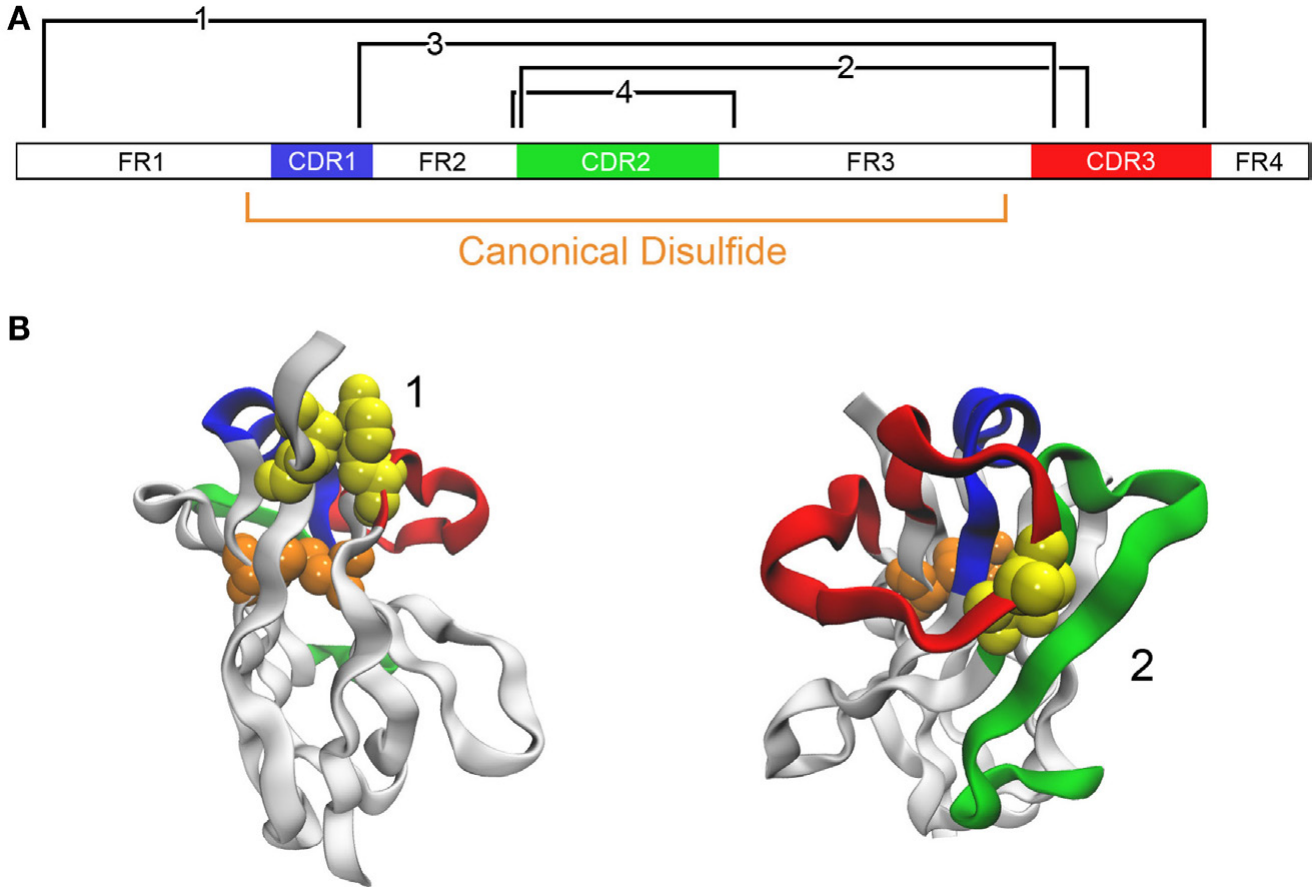
Addition of disulfide bonds to stabilize an antibody. **(A)** The domain structure of a VHH A3 is schematically shown (46). The canonical disulfide bond is formed between Cys located at positions 23 and 104. Locations where it is predicted that disulfide bonds may be introduced are shown in black (Cys introduced at positions 3 and 117 for bond 1, 55 and 111B for bond 2, 38 and 110 for bond 3, and 54 and 78 for bond 4). **(B)** Examples of two candidates are shown with the same color coding. The native residues that are to be mutated to cysteine are shown as a yellow space-filling model. The model uses A3 with PDB code 4TYU.

In work of a similar nature, we examined four locations for the insertion of a second disulfide bond in a llama VHH that already had an impressive *T*_m_ of ~84°C (46) (Figure 3). In addition to the disulfide bond between a pair of Cys introduced at positions 54 and 78, analogous to the one added by previous groups, cysteine residues were engineered at positions 38 and 110 (to promote disulfide bond formation between CDRs 1 and 3), at 55 and 111B (for a disulfide bond linking CDRs 2 and 3), and at 3 and 117 (for formation of a disulfide bond between FR1 and CDR3). In all cases, the added disulfide bond led to an increase in *T*_m_, with the largest (*T*_m_ > 90°C) afforded by the disulfide bond between Cys 54 and Cys 78. In this case, none of the added disulfide bonds had a significant detrimental effect on affinity.

In addition to elevating *T*_m_, introduction of a disulfide bond linking FR2 and FR3 has been shown to increase protease resistance and stability at acidic pH and in the presence of chemical denaturants (47, 48). For example, engineering a disulfide bond at positions 54 and 78 was shown to increase the *T*_m_ of a VHH by ~17°C when measured at a pH of 5.5 and in the presence of 3 M urea (47). A separate study showed that the same disulfide bond addition generated VHHs that had increased *T*_m_ at both neutral pH and pH 2. These VHHs were also more resistant to pepsin, and additionally, four out of six VHHs showed improved resistance to chymotrypsin (48).

Addition of non-canonical disulfide bonds has proven a robust way to stabilize VHHs. The increases in *T*_m_ resulting from an added disulfide bond tend to be between 4 and 20°C (44, 47–49). One limitation is that addition of an extra disulfide bond as well as even the presence of the canonical disulfide bond has been seen to decrease the stability of the sdAb when exposed to temperatures above its *T*_m_, presumably due to disulfide shuffling or other deleterious chemical reactions that prevent refolding (15). An additional limitation is that in some cases affinity can be negatively affected by disulfide bond introduction, nonetheless, many such stabilized clones retain excellent binding ability. Also problematic is that the expression of VHHs in *E. coli* can suffer upon disulfide bond introduction due to improper disulfide bond formation during the folding process. In this case however, we have ascertained that the addition of helper plasmids that produce disulfide isomerases can serve to mitigate this limitation (49, 50).

### Random Mutagenesis and Stringent Selection

To obtain sdAbs stable enough to perform in harsh environments, stringent selection of sdAb libraries can be employed to enrich sdAbs with desired properties such as protease, heat, or chemical stability. This can be done starting with immune libraries, naive libraries, or alternatively starting with sdAbs that bind target but do not possess the desired stability. When starting with binding sdAbs, sequence diversity is often introduced through random mutagenesis/DNA shuffling into sdAb repertoires to better guarantee the inclusion of strong binders toward desired targets.

Traditional panning methodology, based solely on binding as the selective pressure is likely to lead to the isolation of binding sdAbs; however, it does not guarantee isolation of sdAbs with the desired stability characteristics. This is due to the fact that the CDRs mainly determine the specificity and affinity to target, however, the stability against chemical denaturants, heat, protease and extremes of pH mostly relies on the conserved FR sequences in the variable domains. Small sequence variation in FRs that do not have a significant effect on target binding under mild, physiological conditions can lead to dramatic differences in binding under harsh, stringent conditions. In one study researchers selected VHH sequences binding to *Malassezia furfur*, a fungus responsible for the formation of dandruff, under stringent selection for chemical denaturation stability. This study showed that charges and stable structures due to FR sequences facilitated the stability of VHHs and maintained binding to the fungal targets in shampoo and chemical denaturants (51). The sequence analysis, crystal structure conformation, and point mutation analysis showed that a positively charged residue at position 44, (Arg44/Lys44), located in a well conserved 38–45 loop within FR2 is essential for VHH binding to the fungus in shampoo at high pH, and increasing concentrations of denaturants, guanidine-HCl and urea (51). The stability is likely due to the fact that the positively charged residue at position 44 enhances the electrostatic interaction with the negatively charged molecules present in the medium.

The physicochemical attributes desired for orally administered therapeutic sdAbs are stability in low pH and resistance to various gastrointestinal proteases. Random mutagenesis using error-prone PCR to further vary the binder sequence and the subsequent panning under protease pressure were performed to select protease-resistant VHHs that inhibit *Campylobacter jejuni* (52). The proteolytically stable VHHs consisting of two unique residues, Q3 and V5, from their parental binder sequences are resistant to the digestion by trypsin and chymotrypsin, but not pepsin. To further enhance the resistance to all three proteases, an additional non-canonical disulfide bond was introduced by adding two Cys at positions 54 and 78 as described in the Section “Introduction of Non-Canonical Disulfide Bonds” (48). The resulting mutants have almost 100% resistance to pepsin and chymotrypsin and 50% resistance to trypsin with parental wild-type affinity (52). The Cys54/Cys78 disulfide bond also increased the *T*_m_ at acidic pH, which positively correlates with pepsin resistance.

Sequence variation can also be generated through shuffling DNA fragments derived from multiple starting binders. It was shown that without stringent selection, DNA shuffling improved *in vitro* proteolytic stability of the parental anti-*E. coli* F4 fimbriae VHH to 100% resistance to trypsin and chymotrypsin, and 21% resistance to pepsin and also exhibited a 10-fold increase in affinity (53). The most stable and strongest VHH binder had unique G11 and L24 amino acids within the FR1 compared to the wild-type sequences, however, there was no confirmation of the altering effects of these mutations on the proteolytic stability. To further improve the pepsin resistance of the proteolytically stable VHHs, stringent stability selection conditions could be employed.

**Figure 4:**
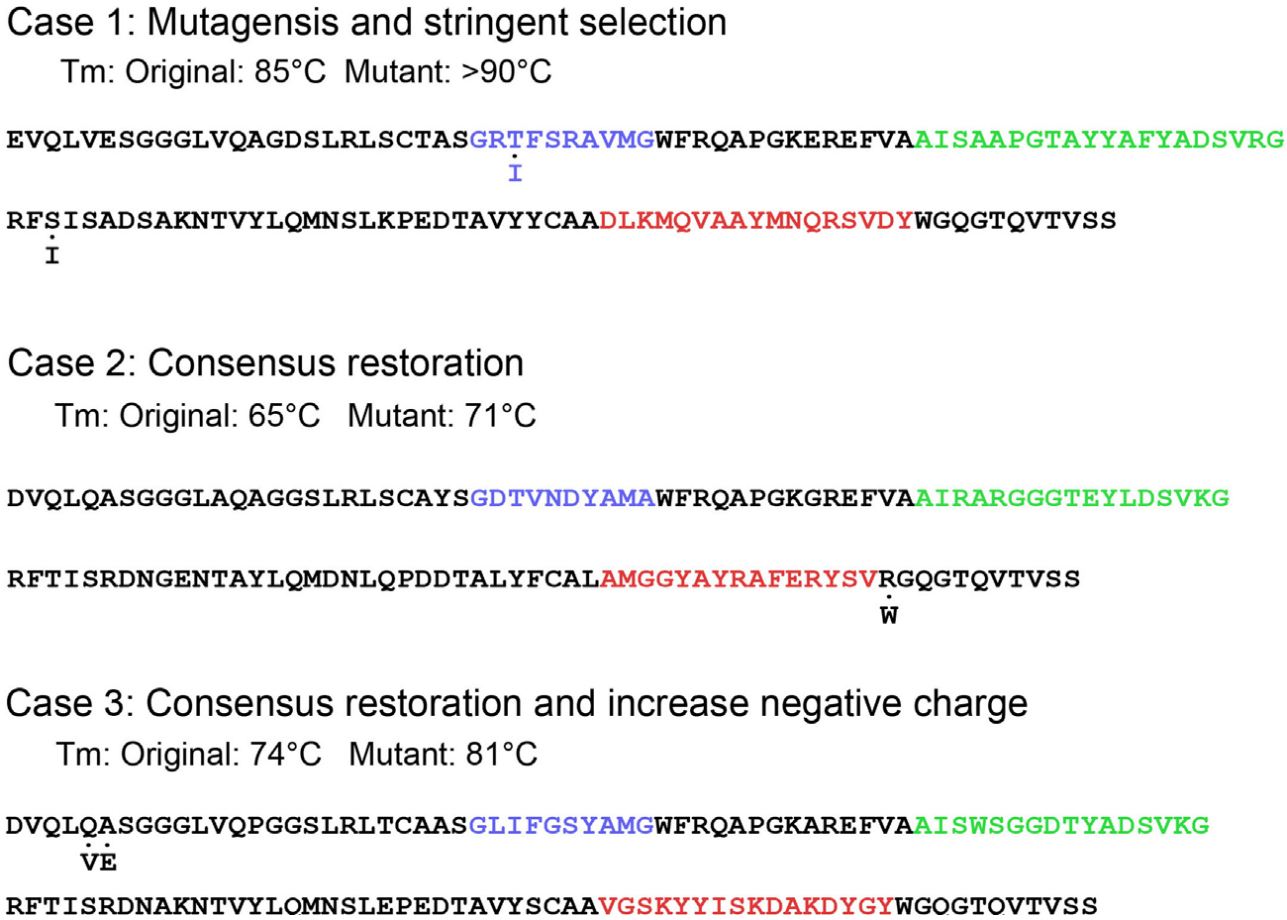
Introduction of point mutations for thermal stabilization. Three case studies in stabilization are illustrated. The original protein sequence is given with color-coding of the complementarity-determining regions. Mutated amino acids are indicated below each sequence.

Although, VHHs already exhibit intrinsically high conformational stability under heat, chemical, and pressure denaturation (22), their physicochemical stability can still be further enhanced using random mutagenesis in conjunction with stringent selection for thermal stability. Our group used a highly thermostable SEB-binding VHH with a *T*_m_ of 84°C as a template to construct a random mutagenesis library and conducted panning under high temperature and high concentration of guanidine-HCl. We found that a clone with two mutated residues, T29I and S77I, further increased the *T*_m_ up to 90°C without compromising the binding affinity to SEB toxin (54) (Figure 4). Like the abovementioned studies with stringent selection, we have also seen that a few amino acid changes within FRs can have significant effects on the physicochemical properties of VHHs.

### Point Mutations

Stability can be engineered into sdAbs by the addition of point mutations (Figure 4). Successful strategies have included the restoration of consensus sequences, substitution of amino acids prone to chemical modification, and changes to the isoelectric points of VHHs.

Although there is variation in the composition of the FR of VHHs, some positions are nearly universally conserved. When binding VHHs are isolated with deviations in these positions, merely restoring the conserved sequence can result in an increase in *T*_m_. For example, ~90% of VHHs contain a Trp at position 118 (20, 55). However, we isolated a ricin-binding VHH that contained an Arg at position 118. Changing Arg to Trp resulted in a 6°C increase in *T*_m_ without compromising the VHH’s affinity (31).

In work examining the mechanism of heat-induced irreversible denaturation of VHHs, researchers assessed the contribution of chemical modification to denaturation by mutating Asn residues in a VHH (14). Three changes were made, one in CDR2 and two in FR regions. In each case the Asn was changed to either Ser or Thr. Although one of the mutations was within a CDR, binding ability was not compromised by the change. Both the wild-type and mutant were subject to continuous heating at 90°C for various times as well as cycling between 90 and 20°C and in each case it was the total time at 90°C that was found to be the critical factor in determining irreversible denaturation. The mutant showed both a small increase in *T*_m_ (~3°C) along with increased functionality on heating to 90°C.

Lowering the isoelectric point is a strategy that has been used to engineer stability and ability to refold after heat denaturation into VHs (56, 57). Introducing mutations that increase the negative charge of VHHs can also improve refolding ability. Three llama-derived VHHs that regained 40–70% of their secondary structure based on CD measurements were subjected to mutagenesis to lower their isoelectric points. In each case, two or three changes were engineered either within FRs or CDRs. In two of the three VHHs, the mutations included changing the neutral wild-type amino acid to Glu or Asp; in the third VHH, two mutations introduced negative charges while the third eliminated a positive charge by mutating a Lys. All three resultant mutants showed marked improvement, being able to refold greater than 95% following heat denaturation (31). The mutations that were used to decrease the charge of these VHHs were located throughout the VHH sequence; in one case the mutations resulted in 5°C decrease in the *T*_m_ and in other cases the *T*_m_s were essentially unchanged.

For VHH variants that fail to refold, increasing their net negative charge such that they experience increased charge repulsion in the unfolded state appears to be sufficient to recover the bulk of the refolding ability of the VHHs. In the case of a VHH that lost the ability to refold on addition of a non-canonical disulfide bond, we found that incorporation of three negative charges within FR1 restored refolding ability and also led to a ~3.5-fold increase in protein expression in *E. coli* without sacrificing affinity or *T*_m_ (58).

Further, we showed that changes within FR1 (Q5V; A6E) of a VHH that lowered the isoelectric point and introduced a sequence observed in several high-*T*_m_ anti-toxin VHH led to a ~7°C increase in the *T*_m_ (58). These changes, incorporated along with other mutations, have also led to increases in *T*_m_ or improved protein expression in *E. coli* in several other VHHs (59). Recently, we demonstrated that a set of changes within FR1 (1E or D, 3Q, 5 V, 6E) of 4 VHHs consistently gave increases in *T*_m_ of 5–9°C, indicating that this might be a general method to increase *T*_m_ of VHHs (60). Independently, two of these changes (1E and 5 V) were identified as stabilizing in a study that examined a large repertoire of sdAb sequences (61).

### Fusion Proteins

One advantage of VHHs is that their ease of expression in many systems facilitates the development of a wide variety of fusion constructs. The vast majority of these constructs have been designed to enhance the utility of VHHs or add additional functionality. For example, their fusion to antiserum albumin sdAbs can extend their serum half-life (62). Fusions to Fc domains or anti-Fc receptor sdAbs can endow them with effector function (63, 64). Fusions with alkaline phosphatase enhances affinity as it homodimerizes and at the same time facilitates the colorimetric detection of targets (65, 66). Fusions with biotin-binding molecules, such as streptavidin or rhizavidin, also form VHH multimers, and allow for the oriented immobilization onto biotinylated surfaces (67, 68). VHH pentamers have also been described, again to take advantage of the binding avidity of multimeric interactions (69).

Comparatively few VHH fusion constructs have been developed with the goal of enhancing stability. In one instance, we utilized the maltose-binding protein (MBP) isolated from the thermophile *Pyrococcus furiosus* to form a VHH-PfuMBP fusion (40). The primary purpose of this fusion was to facilitate production of a VHH in the *E. coli* cytoplasm at higher levels than achievable from the periplasm. A thermostable version of MPB was chosen primarily in order to avoid decreasing the stability of the construct. When produced in the cytoplasm, VHHs often do not form the canonical disulfide bond and hence have lower *T*_m_ than versions of the same VHH produced in the periplasm with an intact disulfide bond. When we measured the *T*_m_ of the VHH portion of the VHH-PfuMBP fusion it was observed to unfold at 68°C which corresponded to the VHH with an intact disulfide bond despite being folded in the cytoplasm. Cytoplasmic production of the unfused VHH showed a lower *T*_m_ (46°C), suggesting that the fusion to MBP enabled the VHH to form its disulfide bond.

In other work, we looked at the effect of adding an α-synuclein tail to the C-terminus of a VHH as a general method for introducing negative charge to increase VHH stability (70). Addition of the negatively charged tail decreased aggregation, increased the ability of several VHHs to bind antigen after heating above their *T*_m_, and restored refolding ability in VHHs that lacked the canonical disulfide bond due to either cytoplasmic expression or mutation of the Cys residues that form the disulfide bond. Impressively, a mutant which lacked the canonical disulfide bond and showed no ability to refold after heat denaturation, was able to regain almost 100% of its secondary structure after heating when expressed with the α-synuclein tail (70). Additionally, we observed that one of the cytoplasmically expressed VHHs with the α-synuclein tail melted at the low temperature associated with lack of the canonical disulfide bond formation, and refolded at the higher temperature observed with an intact canonical disulfide bond. Subsequent heating cycles led to unfolding and refolding both at the higher temperature leading to speculation that the disulfide bond may have formed while the VHH was denatured.

## Stabilizing VNARs

There is much less literature focusing on shark VNARs compared to camelid VHHs. Several recent publications examine stability of VNARs. One detailed the stability of VNARs at extreme pH, in the presence of proteases, and on exposure to elevated temperatures for prolonged periods in liquid, lyophilized, and immobilized formats (32). In another study, the *T*_m_s and refolding ability of VNARs from spiny and smooth dogfish sharks were examined (71).

The first demonstration of engineering stability into a shark VNAR combined strategies of CDR grafting and consensus sequence mutagenesis that had been shown to be effective in raising the stability of VHHs (72). The starting point for this work was a VNAR specific for the nucleoprotein of Ebola virus (73) with a low *T*_m_ (53°C) and a recovery of ~75% of its structure following a single heat denaturation cycle. Two initial graft variants were constructed using a previously identified stable shark VNAR framework. A graft of all 3 CDRs displayed excellent affinity with a low *T*_m_, while a clone where only CDRs 1 and 3 were grafted had poor affinity but a 15°C higher *T*_m_. These two graft variants only had three amino acid differences within CDR2. The CDR2 of shark VNAR (also called HV2) is truncated compared to VHH CDR2. To elucidate which of the amino acids were responsible for the affinity and stability, three double- and three single-point mutants were constructed that covered all the variations between the two graft variants. It was found that a single amino acid change resulted in a 10°C higher *T*_m_ over the original VNAR while maintaining sub-nM affinity equivalent to the original VNAR.

VNARs are gaining popularity as alternatives to VHHs. Protein engineering has been used to increase the affinity of VNARs and there have been efforts to humanize and to improve their pharmacokinetic properties (74–77). It is likely that the other strategies that have been applied to stabilize VHHs and VHs will be tested for their ability to stabilized VNARs as well.

## Concluding Remarks

The availability of stable recognition elements is almost always desirable. Inherently, VHHs and VNARs are generally more robust than conventional recombinant antibody binding domains. However, while these sdAbs are often heat resistant, they are not heatproof. Protein engineering has been applied to VHHs and VNARs to improve their properties. Variants have been produced that are endowed with higher *T*_m_s, greater ability to refold after denaturation, ability to function after heat exposure, and increased tolerance to the presence of chemical denaturants, proteases, and extreme pHs.

We have provided an overview of several methods that have been used successfully to enhance the stability of VHHs and VNARs. Each method, summarized in Table 1, has been used successfully to improve stability, but the extent of improvement varies and needs to be determined empirically. Each method has its benefits and liabilities; the addition of a non-canonical disulfide bond always guarantees at least a few degrees increase in *T*_m_ but can compromise affinity, specificity and/or protein expression in *E. coli*. The strategies for improving sdAb stability can be combined for better results. For example, negatively charged amino acids have been introduced into constructs that also contained an added non-canonical disulfide bond to provide additional increase in *T*_m_ (59). In another instance, non-canonical disulfide bonds were used along with stringent stability selection to develop sdAbs resistant to multiple proteases (52).

Greater understanding of the mechanism of sdAb stability can potentially lead to more general and predictable methods to increase sdAb robustness. These advances will further increase the utility of sdAbs in medical, industrial and biotechnological applications.

## Author Contributions

EG, JL, DZ, and GA outlined, drafted, and revised the manuscript.

## Conflict of Interest Statement

The authors declare that the research was conducted in the absence of any commercial or financial relationships that could be construed as a potential conflict of interest. The reviewer, JT, and handling Editor declared their shared affiliation, and the handling Editor states that the process nevertheless met the standards of a fair and objective review.
